# A Simple Tool for Diet Evaluation in Primary Health Care: Validation of a 16-Item Food Intake Questionnaire

**DOI:** 10.3390/ijerph110302683

**Published:** 2014-03-04

**Authors:** Katri Hemiö, Auli Pölönen, Kirsti Ahonen, Mikko Kosola, Katriina Viitasalo, Jaana Lindström

**Affiliations:** 1 Department of Chronic Disease Prevention, National Institute for Health and Welfare, P.O. Box 30, FI-00271 Helsinki, Finland, E-Mails: mikko.kosola@gmail.com (M.K.); jaana.lindstrom@thl.fi (J.L.); 2 Development of Work and Organizations, Finnish Institute of Occupational Health, Topeliuksenkatu 41 a A, FI-00250 Helsinki, Finland; 3 The Prevention Project of Diabetes and Cardiovascular Diseases, Pirkanmaa Hospital District, P.O. Box 2000, FI-33521 Tampere, Finland; E-Mail: auli.polonen@pshp.fi; 4 Tampere University Hospital, P.O. Box 2000, FI-33521 Tampere, Finland; E-Mail: kirsti.ahonen@pshp.fi; 5 Finnair Health Services, IF/67, 01053 Finnair, Finland; E-Mail: katriina.viitasalo@finnair.com

**Keywords:** food intake questionnaire, nutrition assessment, primary health care, validation studies

## Abstract

Our aim was to validate a 16-item food intake questionnaire (16-FIQ) and create an easy to use method to estimate patients’ nutrient intake in primary health care. Participants (52 men, 25 women) completed a 7-day food record and a 16-FIQ. Food and nutrient intakes were calculated and compared using Spearman correlation. Further, nutrient intakes were compared using kappa-statistics and exact and opposite agreement of intake tertiles. The results indicated that the 16-FIQ reliably categorized individuals according to their nutrient intakes. Methods to estimate nutrient intake based on the answers given in 16-FIQ were created. In linear regression models nutrient intake estimates from the food records were used as the dependent variables and sum variables derived from the 16-FIQ were used as the independent variables. Valid regression models were created for the energy proportion of fat, saturated fat, and sucrose and the amount of fibre (g), vitamin C (mg), iron (mg), and vitamin D (μg) intake. The 16-FIQ is a valid method for estimating nutrient intakes in group level. In addition, the 16-FIQ could be a useful tool to facilitate identification of people in need of dietary counselling and to monitor the effect of counselling in primary health care.

## 1. Introduction

A healthy diet, physically active lifestyle and maintaining normal weight constitute the cornerstones of prevention of several chronic diseases. Lifestyle counselling of high-risk individuals has been shown to prevent e.g., the development of type 2 diabetes in a research setting [1,2]. Validated tools which identify high-risk individuals could facilitate the allocation of available health care resources in most effective ways [3]. Nutrition is one of the key targets of lifestyle guidance to prevent chronic diseases. To be effective, dietary counselling should be individualized and based on clients’ former diet. The dietary quality can be assessed using food records, food frequency questionnaires and for some nutrients, with biomarkers. Food records and comprehensive food frequency questionnaires are time-consuming and require sophisticated data management and biomarkers can be too costly, therefore these methods are not as such suitable to be used in primary health care [4]. Furthermore, health care professionals of varying background and training are counselling persons to make lifestyle changes and they need easy-to-use tools to estimate clients’ diet quality. Tools should also be easily interpreted to estimate achieved dietary changes [5].

Several short diet quality questionnaires have been developed and validated. Many of them have been focused on some specific nutrients or food groups [6,7,8,9,10,11,12,13]. Typically they estimate the intake of fruits and vegetables or the intake of fat. Only a few short questionnaires have been aimed to assess a broader range of dietary intake [14,15,16,17,18]. Two studies validated the diet quality questionnaires by assigning scores for the food group frequency answers, with the total score depicting overall diet quality [14,15]. However, the applied scoring methods have weaknesses, e.g. arbitrary choices in relation to questions included, the cut-off values used, and the scoring of different questions [19].

The aim of the present study was to validate a 16-item food intake questionnaire (16-FIQ) and to create a simple method to estimate daily nutrient intake. Ultimately, the aim was to validate a practical tool for estimating and monitoring diet quality in primary health care.

## 2. Experimental Section

### 2.1. Study Design and Population

The 16-item food intake questionnaire was originally developed for and used in the type 2 diabetes prevention programme (FIN-D2D) in Finland [20]. This questionnaire was developed by nutritionists, nurses, and physicians involved with the programme. We conducted the validation study among volunteers from another diabetes screening and prevention implementation programme which took place in the occupational health care of the Finnair airline [21]. In Finland, occupational health care is an important part of the primary health service system, encompassing about 89% of the workforce. Between June 2007 and June 2008 the employees (excluding aircrew) who came to health check-up that included questionnaires, laboratory tests and measurements were also asked to take part in the validation sub-study. The participants were asked to fill in the 16-item food intake questionnaire and they were advised how to keep food record for 7 days starting the next day after the health check-up. 

Altogether 98 employees were invited, 89 agreed to take part in the study, and finally 77 employees, 52 men and 25 women, completed both the 16-FIQ and the food record. The characteristics of the participants are shown in Table 1. There were no significant differences in characteristics between men and women and between participants and those who dropped out.

All participants gave their written informed consent. The study was approved by the Coordinating Ethics Committee of Hospital District of Helsinki and Uusimaa.

**Table 1:** Characteristics of the participants in 16-item food intake questionnaire validation study (mean (SD) or %).

Characteristic	Men (n = 52)	Women (n = 25)	*p*
Age	45.0 (9.5)	42.7 (8.0)	0.3
Cohabitant (%)	78	68	0.3
Education high (%)	12	16	0.8
Sedentary lifestyle (%)	20	24	0.7
Smoking (%)	15	16	0.1
BMI (kg/m^2^)	26.9 (3.9)	26.5 (4.4)	0.7
Total cholesterol (mmol/L)	5.2 (1.0)	5.0 (0.8)	0.4
fP-Glucose (mmol/L)	5.5 (0.4)	5.4 (0.5)	0.2
Triglycerides (mmol/L)	1.4 (0.7)	1.2 (0.6)	0.3

### 2.2. 16-item Food Intake Questionnaire

The participants were instructed to consider their usual diet during the past month while answering the questions in the questionnaire. The original purpose of the questionnaire was to evaluate the intake of foods and nutrients which are known to affect the risk of type 2 diabetes, specifically the quality of dietary fat and the use of fibre-rich foods. The questionnaire items had been chosen based on the contribution of foods to nutrient intakes according to the results of 2007 48-h recalls in Findiet survey in Finland [22]. 

The questionnaire consists of 16 questions of three question types (see Supplementary materials). Six questions are about number of meals per day and frequency of consumption of fast foods, fruits, vegetables, sugar rich foods, and sweets, with serving size specified in the question. Four questions are about fat or cream used for cooking, fat used on bread, and the type of salad dressings. The rest of the questions are open-ended: number of different dishes per week (fish, sausage, chicken, meat, vegetable), milk in decilitres, cheese in slices, and cold cut products in slices grouped by the fat content, bread and breakfast cereals in slices or decilitres grouped by the fibre content, and beverages in cups, classes or bottles grouped by sugar or alcohol content. 

The nutrient intake estimates of fat, saturated fat, protein, carbohydrate, sucrose, fibre, alcohol, vitamin D, vitamin C, calcium, and iron were estimated from the questionnaire by multiplying the frequency of the food consumption by weight of an estimated average portion and nutrient content of the food in question. If the questionnaire item covered several foods, then average combination of the foods was used. The daily consumption of spread on bread was estimated by multiplying an average portion of spread by the amount of bread slices if the person reported using spread on bread. The fat used for cooking was estimated by the amount of meals eaten per week. If the person reported using salad dressing, one average portion was added in the calculated daily food intake. Finally, energy contents (kJ) were calculated by multiplying fat grams by 37, carbohydrate, sucrose and protein grams by 17 and alcohol grams by 29. The energy from each macronutrient was divided by the total energy and multiplied by 100 for final energy percentage (E%).

### 2.3. Food Records

The participants were asked to keep a food record for seven consecutive days, beginning the following day after the health check-up. All participants were advised by the study nutritionist to write down in detail all the foods and beverages they consumed. A picture booklet of typical foods and a scale were given to the participants to help them estimate portion sizes [23]. The participants could contact the study nutritionist for further advice if needed. The food records were returned by mail and the nutritionist called the participants to ensure the content of food record more precisely, when needed.

Daily nutrient and food intakes were calculated using in-house software based on the Finnish food composition database with 1,229 foodstuffs and beverages and 2,177 recipes of composite dishes [24]. Recipes were modified individually based on the food records. The intake was calculated for the following food groups: fish, sausage, chicken, meat and vegetable dishes, fast foods, vegetables, fruits and berries, milk products, rye bread, multigrain bread, white bread, porridge, breakfast cereals, cheeses, cold cuts, desserts, sweets, tea, coffee, soft drinks, sugar-sweetened juices, fruit juices, beer, wine, and distilled spirits. The daily intakes of energy, fat, saturated fat, protein, carbohydrate, sucrose, fibre, calcium, vitamin D, vitamin C, iron, and alcohol were calculated.

### 2.4. Statistics

The study results were analysed in two phases. First, food and nutrient intakes calculated from food intake questionnaire and food records were compared. Second, the models to estimate nutrient intakes based on questionnaire answers were created. 

Food intake in grams estimated from 7-day food records and food intake frequencies from the 16-FIQ were compared using Spearman correlation (r_s_). Nutrient intakes estimated from the food records and nutrient intakes estimated from the 16-FIQ were compared using Spearman correlation. Significance level was set at α = 0.05. Furthermore, study population was divided into tertiles according to estimated nutrient intakes, and exact and opposite agreement between the methods were calculated along with weighted kappa statistic [25] .

In addition, to be able to give an estimate of the average daily intake of fat, saturated fat, sucrose, fibre, iron, vitamin C, and vitamin D using the questionnaire, simple linear regression models were created. In the modeling, nutrient intake estimates from the food records were used as the dependent variables and sex and sum variables derived from the questionnaire were used as the independent variables. Questionnaire items presumed to have substantial contribution to the intake of nutrient in question were included in the sum variable. Each questionnaire item was then weighted with a multiplier. Multipliers were determined in a loop where it was tested if increasing or subtracting multiplier of the questionnaire item by one increased the coefficient of determination (R^2^) of the model. If R^2 ^increased then multiplier was further increased/subtracted until R^2 ^did not increase anymore. The next questionnaire item was handled in the similar way. This loop was worked through the questionnaire items until none of the multipliers in the model changed. When necessary, response variable was base *e* log-transformed to improve normality and homoscedasticity of residuals. Back-transformation of logarithmic values after model fitting introduces bias into estimates. This is corrected by including multiplicative bias correction factor exp(MSE/2), where MSE is mean squared error, in the models [26]. The analyses were done using the SAS statistical package (SAS Institute Inc., Cary, NC, USA, version 8.2) and R version 2.13.1 [27].

## 3. Results and Discussion

### 3.1. Nutrient Intakes Estimated from the 7-day Food Records

The average energy intake from fat was 33.4%, saturated fat 12.6%, and sucrose 8.7% in men, and in women 33.9%, 12.7%, and 8.6%, respectively. In men the intake of fibre was 21.8 g, vitamin D 6.5µg, vitamin C 80 mg, and iron 13.6 mg. In women the intake of fibre was 17.7 g, vitamin D 5.3 µg, vitamin C 93.5 mg, and iron 10.8 mg.

### 3.2. Food Group Correlations

Spearman correlation coefficients (r_s_) for 26 different food groups ranged from 0.08 for poultry dishes to 0.74 for wine (Table 2). Good correlations were found for milk products (r_s _= 0.68), fruits and berries (r_s_ = 0.65), porridge (r_s_ = 0.65), breakfast cereals (r_s_ = 0.63), beer (r_s_ = 0.61), rye bread (r_s_ = 0.59), desserts (r_s_ = 0.57) and fish dishes (r_s_ = 0.57). Of the food groups, r_s_ was higher than 0.5 for 46%. Use of sausage dishes, poultry dishes and fast foods did not correlate significantly between the two methods. 

**Table 2:** The Spearman correlation coefficients for food group intakes between estimated food intake in grams from the food records and the food intake frequencies from the 16-item food intake questionnaires.

Food Group	Spearman Correlation Coefficients	*p*-value
Fish dishes	0.57	<0.0001
Sausage dishes	0.14	0.24
Poultry dishes	0.08	0.5
Meat dishes	0.28	0.016
Vegetable dishes	0.35	0.002
Fast foods	0.13	0.25
Vegetables	0.36	0.002
Fruits and berries	0.65	<0.0001
Milk and milk products	0.68	<0.0001
Rye breads and crispbreads	0.59	<0.0001
Multigrain breads	0.29	0.01
White breads	0.42	0.0001
Porridges	0.65	<0.0001
Breakfast cereals	0.63	<0.0001
Cheeses	0.39	0.0004
Cold cuts	0.46	<0.0001
Desserts *	0.57	<0.0001
Sweets and sugar	0.48	<0.0001
Tea	0.75	<0.0001
Coffee	0.58	<0.0001
Soft drinks	0.49	<0.0001
Sugar-sweetened juices	0.42	<0.0001
Fruit juices	0.51	<0.0001
Beer	0.61	<0.0001
Wine	0.74	<0.0001
Distilled spirits	0.33	0.022

Note: * Include sweet bakery, milk desserts, ice-cream and chocolate.

Use of fat spreads reported on the food records was regrouped to two categories according to the amount of saturated fats in the spreads. The 16-FIQ correctly classified 86% of those who used vegetable margarines, 67% of those who used butter and butter spreads and 67% of those who did not use fat spreads on bread. 

### 3.3. Nutrient Correlations

Statistically significant correlations between the 16-FIQ and the food records ranged from 0.29 for iron to 0.68 for alcohol (Table 3). 

**Table 3:** The Spearman correlation coefficients and weighted kappa between intakes estimated from the food records and the 16-FIQ and percentage of participants classified into the exact and opposite tertiles of nutrient intakes with the two methods.

Nutrient	Spearman Correlation Coefficient		Agreement (%)		Weighted Kappa
	r_s_	*p*-value	Exact	Opposite	
Energy	0.30	0.005	39.0	14.3	0.15
Fat (E%)	0.37	<0.001	45.5	10.4	0.27
Saturated fat (E%)	0.48	<0.001	44.2	9.1	0.27
Protein (E%)	0.40	<0.001	45.5	13.0	0.24
Carbohydrate (E%)	0.56	<0.001	55.8	7.8	0.41
Sucrose (E%)	0.61	<0.001	57.1	6.5	0.44
Fibre (g)	0.53	<0.001	48.1	7.8	0.33
Alcohol (E%)	0.68	<0.001	57.1	5.2	0.50
Vitamin D (μg)	0.47	<0.001	51.9	9.1	0.35
Vitamin C (mg)	0.53	<0.001	54.5	6.5	0.41
Calsium (mg)	0.32	0.004	40.2	10.4	0.21
Iron (mg)	0.29	0.01	45.5	15.6	0.21

High correlation between the methods was found for sucrose (r_s_ = 0.61), carbohydrate (r_s_ = 0.56), fibre (r_s_ = 0.53), vitamin C (r_s_ = 0.53) and vitamin D (r_s_ = 0.47). Food record and questionnaire classified at least 50% of the participants into the same third of intake for carbohydrate, sucrose, vitamin C, vitamin D and alcohol. Of the participants, between 5.6% and 15.6% were misclassified into the opposite third of intake for different nutrients. The weighted kappa values were in agreement with the r_s_.

### 3.4. Tool for Estimation of Average Daily Nutrient Intakes

**Figure 1:**
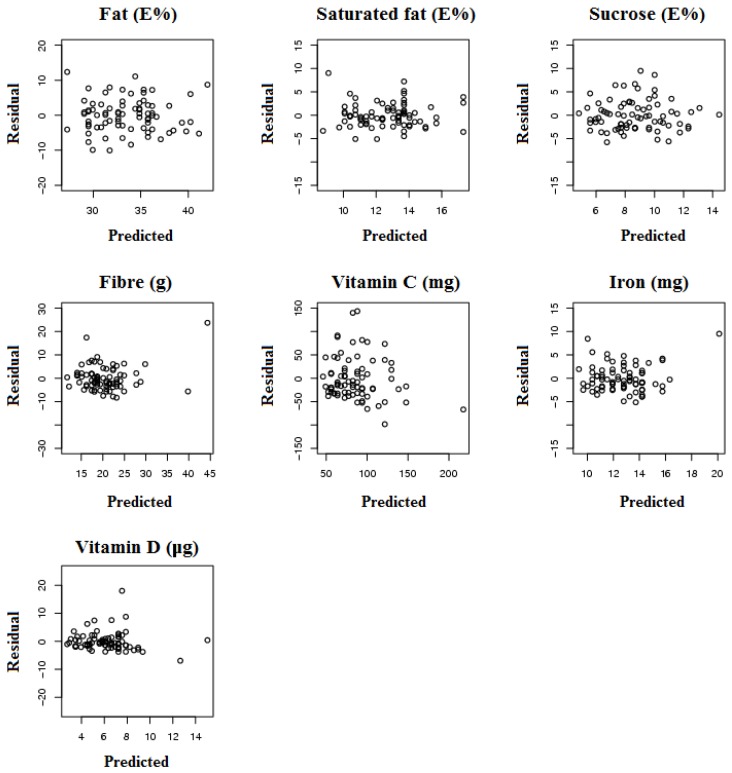
Residuals plotted against nutrient intakes predicted with the regression models.

Linear regression models for estimating daily nutrient intakes from the questionnaire answers were created for total fat (E%), saturated fat (E%), sucrose (E%), fibre (g), vitamin C (mg), iron (mg) and vitamin D (μg) (Table 4). The R^2^ was the highest for fibre (0.49) and the lowest for vitamin C (0.24). Figure 1 shows residuals for these models plotted against the estimated nutrient intakes. 

**Table 4:** The scoring of the food intake questionnaire items based on the linear regression models for predicting nutrient intakes, and instructions **^a^** and an example **^b^** how to count fat intake (E%) based on questionnaire answers.

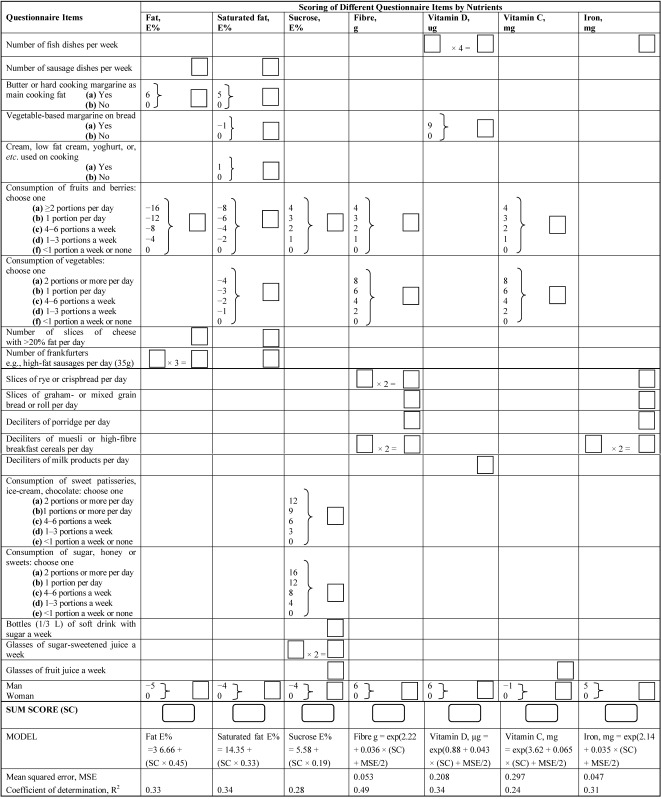

Notes: ^**a**^ Instructions how to fill in the Table 4. Fill in your answer for each question in the white box on the right side of the columns. Complete all the boxes. If two white boxes are on the same row and column, then fill in the answer for the question in the first box and multiply it with the corresponding multiplier and write the points in the second box. Parenthesis means that you need to choose one of the questionnaire answers and write the corresponding points in the box on the right side of the column. Sum points by columns and write the result in the last white box of the columns (“sum score”). **^b^** An example how to estimate fat intake. Male participant’s questionnaire answers are: 2 sausage dishes/ week give 2 points; no use of butter for cooking gives 0 points; 1 portion of fruits and berries/ day gives −12 points; 6 slices of cheese/day give 6 points; no frankfurters gives 0 × 3 = 0 points; male gender −5 points. Fat sum score (SC) is 2 + 0 + (−12) + 6 + (0 × 3) + (−5) = −9, Model to estimate fat intake: 36.66 + (SC × 0.45) = 36.66 + (−9 × 0.45) = 32.6 E%. Estimated fat intake is 32.6 E%.

## 4. Discussion

We evaluated the validity of a short food intake questionnaire containing 16 questions to estimate daily food and nutrient intake. First, we compared the consumption of certain food items estimated with two methods, the 16-item food intake questionnaire and the 7-day food record, to explore whether individuals are able to recall and report their usual intake of these foods accurately. Second, we compared the nutrient intakes estimated with these two dietary analysis methods, to determine whether as few as 16 questions covering selected foods can be used to categorize individuals according to nutrient intakes. Third, we created regression models to give an estimate of persons daily nutrient intakes based on the answers given in the 16-item food intake questionnaire, using the food record data as reference. Our results indicate that the questionnaire gives a reasonably valid estimate of the intake of several food groups and can be used to estimate the intake of fat, saturated fat, sucrose, fibre, vitamin C, vitamin D, and iron with the created regression models. 

Our results are comparable to other validation studies where food group correlations, either Spearman or Pearson, between short food intake questionnaire and reference method (food record, diet recall or diet history) have been studied [15,16,18]. Osler and Heitmann found highest correlations for milk products (r_s_ = 0.56−0.75) and fruits and berries (r_s_ = 0.50–0.66), in parallel to our results, and the lowest for rye and light bread [16]. Also Svilaas *et al*. found high weighted kappa coefficient 0.71 for milk products [15]. For cheese, correlation has generally been lower than for milk products [15,16,28]. Possibly, the amount of cheese in grams is more difficult to estimate than the amount of milk products in household measures. The intakes of fruits and berries are typically in good concordance in the validation studies of short food intake questionnaires [6,9,12,16,28,29]. In our study, correlation for vegetables was 0.36 which was only slightly lower than in the other studies (r_s_ = 0.43–0.55) [9,10,11,16,28]. Therefore, it seems possible to estimate the intake of vegetables rather well with a few questions. Higher correlations were found for fish dishes, rye bread, white bread and desserts in present study than in the other studies [16,18,28]. Low correlations (<0.2) for sausage dishes, poultry dishes and fast foods may result from the low variation, for example all participants in our study reported consuming fast food one to three times a week or less.

The nutrient intakes estimated from the 16-item food intake questionnaire and the food records showed good agreement, with r_s_ exceeding 0.50 for carbohydrates, sucrose, fibre, vitamin C, and alcohol. These results are in accordance with other validation studies [12,17]. Originally, the questionnaire was not developed to assess total energy intake and therefore several food groups that contribute significantly to energy intake (staple foods such as boiled potatoes, rice, and pasta) were left out. Consequently, the estimation of total energy was not very accurate (r_s_ = 0.30). Also some other nutrients, such as iron and calcium that were not the focus of the questionnaire have low agreement between the two methods. The short food intake questionnaires are most reliable when only a limited number of nutrients are estimated. 

The question whether the created regression models can be used to estimate individual’s nutrient intake and target nutrition counselling is important to explore. Food frequency validation studies have commonly measured agreement between two methods using correlation coefficients and the Bland-Altman method [30]. However, in our study the correct use of Bland-Altman method would have required cross-validation of the models in a separate data set which due to small sample size was not possible. Instead, we illustrated the performance of the models by plotting the residuals against the nutrient intakes estimated with the models. It should be noted that these plots overestimate the performance of the models because plots include same individuals that were involved in model construction. The figures show that the models predict fibre, iron and vitamin D rather similarly compared with nutrient intakes estimated from the food record. The result is what we expected as the major food sources of these nutrients are few and thus it is possible to estimate the level of nutrient intake with short questionnaire at individual level. The predictive models of fat, saturated fat, sucrose and vitamin C intake show broader range of residuals and thus are not well suited for accurate estimation of individual intake, but could be used to estimate group level intakes.

Interestingly, in this population the amount of sausage dishes predicted fat and saturated fat intake in regression models even though the amount of sausage dishes compared between food record and the questionnaire did not correlate well. Presumably, reporting frequent consumption of sausage dishes may express persons’ dietary pattern in a broader sense. Other studies using factor analysis have shown that persons who eat processed meat including sausages typically have higher fat intake [31,32]. 

The strength of our study is that we used the food record as the reference method, as recommended [33]. Furthermore, collecting data with a food record does not depend on respondents’ ability to remember and perceive usual diet for a long time contrary to food frequency questionnaires or diet history methods. The difficulty of food recording is how to estimate portion sizes accurately. We gave participants a scale and a picture booklet which have been demonstrated to be a useful aid for the quantification of most food items [34]. Keeping a food record might have induced changes in participants’ diet and it is known that usually the change is towards more healthy diet. However, the relatively good agreement (r_s_ = 0.48–0.57) between the two methods regarding foods that are considered unhealthy, such as desserts, sweets and sugar, and soft drinks indicates that diet did not change much during the recording period. 

The present study has limitations that need to be addressed. All participants lived in an urban area and therefore the results cannot be generalized to people living in a rural area where food habits may differ from those in cities. Another limitation is that compared with general Finnish population the participants were more educated. Even though this may not affect the results of the validation study because the comparisons were made intra-individually it should be kept in mind that the results may not apply to a lower educated population. Therefore, if the questionnaire is used in a very different kind of population or in another country where local habits and food culture are different its validity should be confirmed.

The study participants were asked to start keeping the food record the morning following the health check-up that included the food intake questionnaire. The food recording immediately after the completion of the questionnaire might have influenced the participants’ food choices. A clearance period between the two methods would have been advisable but was not possible as the participants were given dietary counselling soon after the health check-up, according to the main study protocol (prevention of type 2 diabetes) ,which might have affected the participants’ eating habits. For the same reason we could not study the reproducibility of the questionnaire in this study.

The 16-FIQ was developed to help the lifestyle counsellors in primary health care to identify patients whose food choices do not refer to a healthy diet. In this paper we describe a method to estimate nutrient intake from the 16-FIQ. In practice the questionnaire with the nutrient intake estimation equations can be easily computerized and patients could fill in the questionnaire online in a few minutes. In addition, patients could get a feedback and instructions about how to change their food choices towards healthier ones. The effect of counselling on diet changes can be evaluated with the questionnaire and the information can be used to monitor and improve nutrition counselling in primary health care. 

## 5. Conclusions

In conclusion, the 16-item food intake questionnaire measures intake of several food groups rather well and could be a useful tool for identifying persons at need for nutrition counselling. In addition, the questionnaire could be used to estimate nutrient intakes at group level, and thus facilitate monitoring the effectiveness of dietary counselling in primary health care.

## Supplementary Files

Supplementary File 1 Supplementary Information (PDF, 126 KB)
