# Supplementary material for: A Simple Tool for Diet Evaluation in Primary Health Care: Validation of a 16-Item Food Intake Questionnaire
Source: Int J Environ Res Public Health. 2014 Mar 4;11(3):2683–97. doi: 10.3390/ijerph110302683 (PMC3986998; doi:10.3390/ijerph110302683)
Supplement: Supplementary File 1 — Supplementary Information (PDF, 126 KB) [file ijerph-11-02683-s001.pdf]

Supplementary Information

Simple Tool for Diet Evaluation in Primary Health Care: Validation of 16-Item Food Intake Questionnaire

---

1. How many meals and snacks in all do you usually eat during weekday? Snack is e.g., fruit, chocolate bar, sandwich, juice, beer.

1. 1–2 meals and snacks
2. 3–4 meals and snacks
3. 5–6 meals and snacks
4. 7 or more

2. How many servings a week do you eat the following dishes as a main course?

1. Fish dishes ______ times (e.g., baked fish, fried Baltic herrings, fish soup, herring, cured salmon)
2. Sausage meals ______ times (e.g., baked sausage, sausage soup, sausage stew)
3. Poultry dishes______ times (e.g., grilled chicken, chicken fricassee, chicken salad)
4. Meat dishes______ times (e.g., meat soup, steak, meatballs, pork chop, liver casserole)
5. Vegetarian dishes______ times (e.g., vegetable soup, spinach pancake, vegetable salad)

3. How often do you eat fast food?

1. Serving or more per day
2. 4–6 servings a week
3. 1–3 servings a week
4. 1–3 servings a month
5. Less than 1 serving a month or none

4. What type of cooking fat or oil is most often used in your household?

1. Vegetable oil or liquid margarine
2. Vegetable margarine with 60-80% fat
3. Vegetable sterol margarine
4. Hard cooking margarine
5. Butter-vegetable oil mixture
6. Butter
7. Nothing/ we do not cook

5. What type of cream is most often used in your household?

1. Cream-vegetable oil mixture
2. Yoghurt for food preparation
3. Low fat cream, low fat crème fraîche, low fat sour cream, cultured half cream
4. Cream, crème fraîche, sour cream
5. Nothing/we do not cook

6. How much do you eat vegetables? One portion is e.g., 1 decilitre of cooked or root vegetables, 1 medium-sized carrot or 2 tomatoes?

1. 2 portions or more per day
2. 1 portion per day
3. 4–6 portions a week
4. 1–3 portions a week
5. Less than 1 portion a week or none

7. How much do you eat fruit and berries? One portion is medium-sized fruit or 2 deciliters of berries?

1. 2 portions or more per day
2. 1 portion per day
3. 4−6 portions a week
4. 1−3 portions a week
5. Less than 1 portion a week or none

8. What kind of salad dressing do you usually use?

1. Nothing
2. Vegetable oil or oil-based dressing (french dressing, mayonnaise etc.)
3. Juice-based dressing
4. A dressing based on cultured half cream or yogurt

9. How many deciliters of milk or liquid milk products do you consume per day? 1 glass = 2 dL

1. _____ deciliters of milk products with <1% fat (skimmed milk or fat-free yogurt)
2. _____ deciliters of milk products with 1%–2% fat (semi-skimmed milk)
3. _____ deciliters of milk products with 2%–3% fat (full fat milk or regular yogurt)
4. _____ deciliters of milk products with >3% fat or more
5. I do not usually consume milk products

10. How much do you on average eat bread and other cereals per day?

A slice = a ready cut slice or half of a roll

1. ______ slices of rye- or crispbread
2. ______ slices of graham- or mixed grain bread or roll
3. ______ slices of white bread or roll
4. ______ deciliters of porridge (e.g., rye-, oat- or wheat flake porridge)
5. ______ deciliters of low-fibre breakfast cereals (e.g., corn flakes or rice crispies)
6. ______ deciliters of muesli
7. ______ slices of sweet bread

11. What kind of spread do you usually use on your bread?

1. Reduced-fat margarine (28%–60% fat)
2. Soft margarine with 70%–80% fat
3. Vegetable sterol margarine
4. Butter-vegetable oil mixture
5. Butter
6. Nothing

12. How much do you usually eat cheese per day? A slice of cheese is about 10 g.

1. ______ slices of cheese with <20% fat
2. ______ slices of cheese with >20% fat
3. ______ slices of cheese with vegetable fat
4. Less than a slice per day or none

13. How much do you usually eat cold cuts per day? A slice is about 10 g.

1. _______ slices of cold cuts with <10% fat
2. _______ slices of cold cuts with >10% fat
3. _______ pieces of frankfurter (35g) *etc.*
4. _______ less than a slice per day or none

14. How much do you eat sweet patisseries, ice cream, puddings or chocolate? One portion is e.g., a piece of pie or cake, a small doughnut or Danish pastry, 3–4 cookies, ice cream cornet, pudding, chocolate bar.

1. 2 portions or more per day
2. 1 portion per day
3. 4−6 portions a week
4. 1−3 portions a week

5. Less than 1 portion a week or none

15. How much do you eat sugar, honey or sweets? On portion is e.g., 2 teaspoons of sugar or honey, 3 sugar lumps, 5 sweets.

1. 2 portions or more per day
2. 1 portion per day
3. 4–6 portions a week
4. 1–3 portions a week
5. Less than 1 portion a week or none

16. How much on average do you drink the following beverages a week? Mark 0, if less than once a week.

1. _______ cups of regular tea (1 cup = 2 dL)
2. _______ cups of regular coffee (1 cup = 1 dL)
3. _______ bottles of soft drink with sugar (1 bottle = 1/3 L)
4. _______ bottles of sugar-free soft drink (e.g., Coca Cola Light)
5. _______ glasses of fruit juice (1 glass = about 2 dL)
6. _______ glasses of sugar-sweetened juice
7. _______ bottles of beer, cider etc. (1 bottle = 1/3 L)
8. _______ bottles of beer, cider etc. (1 bottle = 1/2 L) or strong beer (1 bottle = 1/3 L)
9. _______ glasses of wine (1 glass = 12 cL)
10. _______ portions of spirits (e.g., vodka, whisky, gin, cognac, liquers *etc*. 1 portion= 4 cL)

© 2014 by the authors; licensee MDPI, Basel, Switzerland. This article is an open access article distributed under the terms and conditions of the Creative Commons Attribution license (http://creativecommons.org/licenses/by/3.0/).
